# Platelet-rich plasma (PRP) treatment of the ovaries significantly improves fertility parameters and reproductive outcomes in diminished ovarian reserve patients: a systematic review and meta-analysis

**DOI:** 10.1186/s13048-024-01423-2

**Published:** 2024-05-17

**Authors:** Máté Éliás, Márton Kónya, Zsófia Kekk, Caner Turan, Isabel Pinto Amorim das Virgens, Réka Tóth, Márton Keszthelyi, Péter Hegyi, Szabolcs Várbíró, Miklós Sipos

**Affiliations:** 1 Centre for Translational Medicine, Semmelweis University, Budapest, Hungary; 2 Division of Pancreatic Diseases, Semmelweis University, Budapest, Hungary; 3 Institute for Translational Medicine, University of Pécs, Pécs, Hungary; 4 Center of Assisted Reproduction, Semmelweis University, Budapest, Hungary

**Keywords:** Platelet-rich plasma, PRP, Diminished ovarian reserve, DOR, Premature ovarian failure, POF

## Abstract

**Introduction:**

The incidence of infertility caused by diminished ovarian reserve has become a significant problem worldwide. The beneficial effect of PRP treatment of the ovaries has already been described, but the high-level evidence of its effectiveness has not yet been proven.

**Materials and methods:**

A systematic search was performed in five databases, until March 12th, 2024. Both randomized and non-randomized studies that compared PRP treatment of the ovaries to self-control among women with diminished ovarian reserve were eligible for inclusion.

Hormonal levels (Anti-Müllerian hormone (AMH), Follicle stimulating hormone (FSH), Luteinizing hormone (LH), Estradiol (E2), In-vitro fertilization parameters (Antral follicle count, oocyte, and embryo count), biochemical and spontaneous pregnancy and livebirth were measured.

**Results:**

38 eligible studies were identified reporting on 2256 women. The level of AMH rised, the level of FSH decreased significantly after the PRP treatment. AMH 1 month MD 0.20 (*n* = 856, p > 0.001, 95% CI: [0.12;0.28]), 2 months MD 0.26 (*n* = 910, *p* = 0.013, 95% CI: [0.07;0.44]), 3 months MD 0.36 (*n* = 881, *p* = 0.002,95% CI: [0.20;0.52]). FSH 1 month MD -10.20 (*n* = 796, *p* > 0.039, 95% CI: [-19.80;-0.61]), 2 months MD -7.02 (*n* = 910, *p* = 0.017, 95% CI: [-12.48; -1.57]), 3 months MD -8.87 (*n* = 809, *p* = 0.010, 95% CI: [-14.19; -3.55]).

The antral follicle count elevated significantly MD 1.60 (*n* = 1418, *p* =  < 0.001, 95% CI: [0.92; 2.27]). Significant improvement was observed in the number of retrieved oocytes MD 0.81 (*n* = 802, *p* = 0.002, 95% CI: [0.36; 1.26]), and embryos created MD 0.91 (*n* = 616, *p* = 0.001, 95% CI: [0.45;1.36]).

The incidence of spontaneous pregnancy following PRP treatment showed a rate with a proportion of 0.07 (*n* = 1370, 95% CI: 0.04–0.12), the rate of biochemical pregnancy was 0.18 (*n* = 1800, 95% CI: 0.15–0.22), livebirth was 0.11 (*n* = 1482, 95% CI: 0.07–0.15).

**Conclusions:**

Our meta-analysis showed that based on protocolized analysis of the widest scientific literature search to date, containing predominantly observational studies, PRP treatment resulted in a statistically significant improvement in the main fertility parameters of diminished ovarian reserve women. Further multicenter, randomized trials, with large patient numbers and a longer follow-up period are needed to certify our results and develop the most effective treatment protocol.

**Graphical Abstract:**

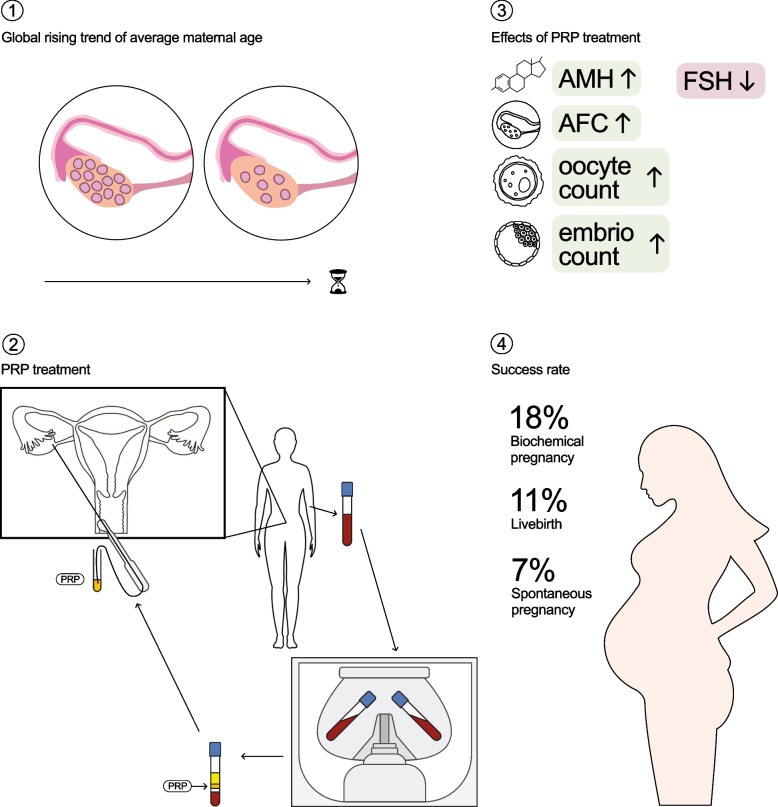

**Supplementary Information:**

The online version contains supplementary material available at 10.1186/s13048-024-01423-2.

## Introduction

About one in six people will experience infertility at some stage in their lives [1]. Furthermore, the global trend towards higher parental age presents certain challenges for specialists in the field of reproduction [2]. One of the main concerns is the observed fertility limitations associated with higher maternal age, which leads to diminished ovarian reserve [3]. Patients with diminished ovarian reserve represent a population with one of the worst prognoses for successful pregnancy, even with assisted reproductive treatments. In these cases, in vitro fertilization (IVF) is the preferred treatment.

For those patients with the worst prognoses for successful pregnancy, egg donation is the only possibility, but this may be unacceptable for many patients for ethical, personal, or financial reasons. Therefore, in the absence of a widely accepted, effective and safe treatment which restores the functioning of the ovaries at least temporarily, more and more people remain childless. Platelet-rich plasma (PRP) treatment of the ovaries can be a promising new treatment method for these patients with poor fertility prognosis.

The main etiological cause of ovarian failure is reduced ovarian function due to a lack of stimulable primordial follicles [4]. PRP treatment is one of the options for enhancing the number of available oocytes.

The angiogenesis promoting molecular network is significantly disrupted in patients presenting with ovarian insufficiency [5]. An interrupted supply of oxygen, nutrients, and hormones is the reason for compromised follicular growth. These conditions appear to be reversible and follicular growth can be stimulated if the compromised ovarian microenvironment is restored [6–8].

A vascular endothelial growth factor (VEGF) mediated ovarian blood flow seems to be a significant factor of compromised folliculogenesis [9–11].

PRP is a fraction of autologous blood plasma concentrated with platelets. Platelets are anucleated cytoplasmic fragments of megakaryocytes differentiated down the myeloid cell lineage [12], containing a complete set of growth factors (PDGF, IGF, VEGF, FGF, TGF-β), coagulation factors, and differentiation factors, which contribute to several angiogenetic, immunosupressive and regeneration processes [13–16].

VEGF is involved in neovascularization through its significant endothelial chemokine and mitogenic effect and a VEGF mediated ovarian blood flow seems to be a significant factor of compromised folliculogenesis [9–11, 17].

PDGF promotes endothelial cell proliferation, playing an important role in angiogenesis [18]. TGF-β regulates the mitogenic effects of other GFs, inhibits macrophage and lymphocyte proliferation, and stimulates the proliferation and differentiation [19] of undifferentiated mesenchymal cells [20]. IGF-1 can reduce the expression of programmed cell death [15].

PRP treatment is a highly promising, new method, successfully applied in several fields of regenerative medicine. PRP was first applied in ovarian rejuvenation by Pantos et al. in 2016 [21], who described a successful temporary ovarian activity restoration in peri-menopausal women after an autologous ovarian platelet-rich plasma treatment. In several observational studies, ovarian PRP treatment improved Follicle stimulating hormone (FSH) and Anti-Müllerian hormone (AMH) levels, enabled spontaneous pregnancies, and even resulted in improved IVF results, as was demonstrated in a clinical trial involving a patient group with very poor prognoses [22]. Despite the promising results, ovarian PRP treatment is still not accepted and therefore, not widespread in clinical practice. This is because reliable clinical evidence for the effectiveness of ovarian PRP treatment is still lacking.

The aim of this study was to collect all relevant clinical data on the effect of ovarian PRP treatment and to summarize the results in order to draw convincing conclusions about its effectiveness.

To the best of our knowledge, this is the widest, and most detailed meta-analysis in this field, the results of which may help the establishment of ovarian PRP treatment in evidence-based clinical reproductive practice.

## Methods

The present systematic review and meta-analysis was carried out conclusively with the PRISMA 2020 guideline [23] Table S3, while the Cochrane Handbook [24] was followed. The study protocol was registered on PROSPERO (registration number CRD42022377931).

### Information sources and search strategy

The systematic search was performed in MEDLINE (via PubMed), Embase, Cochrane Central Register of Controlled Trials (CENTRAL), Web of Science, and Scopus, covering a period from inception until March 12th, 2024. In addition, the reference list of the studies was screened for further eligible articles.

The systematic search was carried out with the following predefined search key: Ovary OR IVF OR In Vitro Fertilization OR POI OR Primary Ovarian Insufficiency OR POF OR Premature Ovarian Failure OR Infertility OR Poor Ovarian Response AND (PRP OR Platelet-rich plasma OR Thrombocyte-rich plasma). Filters or language restrictions were not applied during the search.

### Eligibility criteria

Studies were included, if participants were women aged between 18 and 55 years with diminished ovarian reserve (DOR), premature ovarian failure (POF), or premature ovarian insufficiency (POI), and participants were assessed before and after intra-ovarian injection of PRP. If at least one of the following criteria was true, we considered a patient as a diminished ovarian reserve patient: basal FSH > 15 IU/L, basal anti-Müllerian hormone < 1 ng/ml, antral follicle count < 5, or Bologna criteria of diminished ovarian reserve or POSEIDON criteria 3 or 4 with low prognosis assisted reproductive technology (ART) outcome were met.

According to study design, both randomized and non-randomized studies were eligible for inclusion. Studies without original research data, such as letters to the editor, correspondences, or reviews, were not eligible.

The investigated intervention was PRP injection into the ovaries. All studies were included, in which intraovarian PRP treatment was used, regardless of the count of administration, the administered PRP volume, the method of the preparation and administration of PRP.

Self-control measured before the intervention was considered as a basis for comparison. The main measured outcome was biochemical pregnancy rate (positive pregnancy test or elevated ß-HCG level 2 weeks after embryo transfer). Rate of spontaneous pregnancy, the rate of Livebirth, and other fertility parameters, including Antral Follicle Count (AFC), number of transferable embryos, and mature oocytes were also analysed. In the case of hormonal parameters, the levels of Anti-Mullerian Hormone (AMH), Follicle Stimulating Hormone (FSH), Lutheinizing Hormone (LH), and estradiol (E2) were collected one, two, and three months after the PRP treatment.

Missing time-flow information regarding hormone level measurements was taken as an exclusion criterion. In the case of variable measurement time-points, an aggregate outcome was chosen based on the largest sample size for quantitative synthesis.

In the cases of animal studies, female mammals of all species and ages were included, provided they were treated with PRP against diminished ovarian reserve. Placebo, sham intervention, or no intervention group were the comparators. AMH, FSH and estradiol hormone levels were collected as outcomes.

### Data extraction and quality assessment

Publications were screened based on title and abstract first, and full-text after. Selection was conducted by two independent reviewers (M.É., Z.K.); disagreements were resolved by a third independent reviewer (C.T.).

For data extraction, a standardized data collection sheet was created based on the consensus of methodological and clinical experts. Two independent review authors extracted data from the eligible articles using the standardized data collection sheet; disagreements were resolved by a third independent reviewer.

The following data were extracted from the eligible articles: title, first author, year of publication, study design, main study findings, patient demographics, interventions, outcomes (biochemical pregnancy, spontaneous pregnancy, livebirth, AMH, FSH, AFC, embryo count, count of oocytes). Authors of the included studies were asked for any missing data and elaboration on the reporting whenever needed.

For continuous variables, baseline and after treatment mean and standard deviation (SD) values were extracted. Means and SDs were calculated from case studies if they had at least three fully reported cases. For dichotomous data, events for the outcomes and total numbers of patients were used. For case studies who had multiple IVF cycles before or after the treatment, average counts were used.

Non-randomized studies' risk of bias assessment was undertaken using the ROBINS-I tool [25], for case reports and case series, JBI [26] and for animal studies Syrcle [27], as per the recommendations by the Cochrane Handbook for Systematic Reviews of Interventions [24]. After the risk of bias assessment, two reviewers (É.M., Z.K.) independently assessed the level of evidence certainty using GRADE Pro software [28]. Any and all discrepancies were settled by a third reviewer (C.T.).

### Synthesis methods

All statistical analyses were made with R (v4.3.2) [29] using the meta (v6.5.0) [30], metafor (Viechtbauer 2023, v4.0.0 [31] and clubSandwich [32] packages. All applied models were random-effect meta-analyses. For biochemical pregnancy, livebirth, and spontaneous pregnancy, event proportions were pooled using a generalized linear mixed model with a maximum-likelihood estimator. The rest of the models used a restricted maximum likelihood estimator. For the three animal hormone outcomes, the means of the baseline values in the “control” group and the after-treatment means in the “intervention” group were compared using the metacont function of the meta package, as the two groups were independent in each study. For the hormonal (AFC, FSH, LH, estradiol) and count (AFC, embryo, and oocyte count) outcomes, only self-control data was available. Means were pooled at each time point with at least three studies. Differences between the baseline and those time points were pooled by fitting multivariate models using the metafor package to account for the correlation between before and after measurements on the same patients. Initially, we assumed the same correlation (0.6) between pairs of measurements and used the robust approach of Pustejovsky et al. [32] implemented in the clubSandwich package. In the study published by Keikha et al. [33] only one ovary was treated and the other side was the internal control, so countable outcomes from the treated ovary were doubled to estimate of what the result would be from both ovaries. For outcomes that reported after values for multiple time points (AMH, FSH, LH, estradiol), we also fitted a multivariate model including all time points as a sensitivity analysis. We tried three different variance–covariance matrices for the sampling error, changing according to whether time points closer to each other were assumed to be more correlated, and whether after-treatment measurements were assumed to be less correlated to baseline values than to each other. Each option yielded similar results even before applying the robust correction (Table S2).

Where studies reported quartiles instead of the mean and SD or SE, we used the methodology of Luo et al. [34] and Wan et al. [35] implemented in the meta package to estimate the mean and SD values from quartiles.

Findings of the mean pools and the before-after meta-analyses were visualized on forest plots.

Case series with three or more fully reported cases were added to the meta-analyses, producing separate versions.

Sensitivity analyses were performed for each outcome in the meta-analysis. None of the analyses were found to be eligible for exclusion based on the results.

## Results

### Search results

**Fig. 1:**
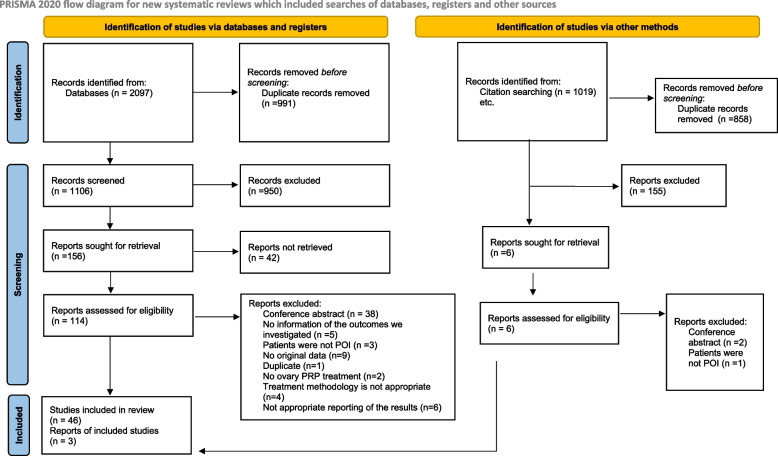
PRISMA flow diagram of selection

During the systematic search, 2097 studies were identified; after the selection process, 49 eligible articles were found. Detailed information about the selection of studies for inclusion is shown in the PRISMA flow diagram Fig. 1.

In the case of human studies, 38 articles were suitable for analysis. According to the study plan, 9 case reports, 4 case series, and 25 observational studies were selected. Characteristics of the included studies are reported in Table 1.

**Table 1:** Baseline characteristics of the included human studies

Author, Publication year	Study type	Number of patients	Age	Duration of infertility (years)	Previous IVF cycles	Number of administration	Administered volume per ovary (ml)
Garavelas A et al., 2023 [36]	prospective observational study	253	43.73 ± 5.89^a^	N.A	N.A	1	4
Najafian A et al., 2023 [37]	prospective observational study	50	39 (35–43)^b^	4 (2–6)^b^	N.A	1	4
Safarova S et al., 2023 [38]	Retrospective observational	60	36.5 ± 0.8^a^	3.8 ± 2.6^a^	N.A	1—3	0.6
Tanha FD et al., 2023 [39]	prospective observational study	20	41.8 ± 1.82^a^	9.7 ± 1.89^a^	N.A	1	3
Tickoo S et al*.*, 2023 [40]	Retrospective observational	66	N.A	N. A	N.A	3	2
Keikha F et al., 2022 [33]	prospective observational study	12	40.04 ± 3.91^a^	2.79 ± 1.79^a^	0.50 ± 0.67^a^	1	4
Barad DH et al., 2022 [41]	prospective observational study	80	44.17 ± 5.45^a^	N.A	≥ 1	1	1.5
Cakiroglu Y. et al., 2022 [42]	prospective observational study	496	40.3 ± 4^a^	7.4 ± 6^a^	N.A	1	3.5
Hosseinisadat R et al., 2022 [43]	prospective observational study	22	33.91 ± 6.58^a^	4.3 ± 4.25^a^	N.A	1	N.A
Rezk MR et al., 2022 [44]	prospective observational study	50	31.1 ± 4.38^a^	2.66 ± 1.33^a^	> 1	1	1
Navali N et al., 2022 [45]	prospective observational study	35	40.43 ± 0.26^c^	N.A	≥ 1	1	2
Parvanov D et al*.*, 2022 [46]	prospective observational study	66	40.5 (34–46)^d^	N.A	2.9 (2–5)^d^	2	0.5
Tülek F et al., 2022 [47]	Retrospective observational	69	38.04 ± 3.86^a^	N.A	N.A	1	2
Aflatoonian A et al*.*, 2021 [48]	prospective observational study	26	34.88 ± 4.5^a^	5.06 ± 1.91^a^	N.A	2	1.5 than 3
Dubinskaya ED et al., 2021 [49]	prospective observational study	52	37.68 ± 7.26^a^	5.07 ± 2.3^a^	2.78 ± 1.15^a^	1	1
Farimani M et al*.*, 2021 [50]	Retrospective observational	72	N.A	N.A	N.A	1	2
Hsu C et al., 2021 [51]	prospective observational study	12	44.42 ± 2.84^a^	13 ± 7.7^a^	N.A	1	3
Pacu I et al., 2021 [52]	retrospective observational study	20	37.4 (31–44)^d^	N.A	> 1	1	2–4
Cakiroglu Y et al*.*, 2020 [53]	prospective observational study	311	34.8 ± 4.3^a^	6.8 ± 4.9^a^	N.A	1	4–8
Melo P et al*.*, 2020 [54]	prospective observational study	46	41(39–44)^b^	N.A	N.A	3	0.2
Petryk N and Petryk M, 2020 [55]	prospective observational study	38	31-45^e^	N.A	> 2	1	0.7
Sfakianoudis K et al., 2020 [22]	prospective observational study	119	41.66 ± 5.66^a^	N.A	N.A	1	4
Sills ES et al., 2020 [56]	prospective observational study	182	45.4 ± 6^a^	≥ 1	≥ 1	1	≥ 1
Abdullah TH et al*.,* 2019 [57]	prospective observational study	50	39.74 ± 7.03^a^	5.2 ± 3.82^a^	N.A	1	1.25
Farimani M et al., 2019 [58]	prospective observational study	12	35.57 ± 3.8^a^	6.5 ± 3.77^a^	4 ± 0.94^a^	1	2
Tremellen K et al*.*, 2022 [59]	Case series	18	40 (35–42)^b^	3 (3–4)^b^	5.05 ± 2.09^a^	1	3
Pantos K et al*.,* 2019 [60]	Case series	3	37.67 ± 7.93^a^	N.A	N.A	1	4
Sfakianoudis K et al., 2019 [6]	Case series	3	38.0 ± 1.41^a^	N.A	12 ± 4.97^a^	1	5
Sills ES et al., 2018 [61]	Case series	4	42.0 ± 4.0^a^	5 ± 2.08^a^	≥ 1	1	1
Shrivastava J et al*.*, 2024 [62]	Case report	1	29	1.5	1	1	1.5–2
Kulakova EV et al., 2022 [63]	Case report	1	34	5	4	2	1.5
Kraevaya EE et al*.*, 2021 [64]	Case report	1	31	2	1	1	0.5
Merhi Z et al*.*, 2021 [65]	Case report	1	33	> 1	0	1	3
Sabouni R et al., 2021 [66]	Case report	1	35	5	0	1	0.5
El Sherbeny MF, 2020 [67]	Case report	1	34	3	2	1	5
Hsu C et al*.*, 2020 [68]	Case report	1	37	4	2	1	2.5
Sills ES et al*.*, 2020 [69]	Case report	1	41	N.A	10	1	≥ 1
Sfakianoudis K et al*.*, 2018 [70]	Case report	1	40	6	0	1	4

*N.A.* Not available, or inappropriate reporting

^a^Mean and Standard deviation

^b^Median and Interquartile range

^c^Mean and Standard error

^d^Mean and Minimum – Maximum

^e^Minimum and Maximum

In case of animal studies 11 articles were eligible for analysis, and all of them was randomized controlled trials by study design as shown on Table 2.

**Table 2:** Baseline characteristics of the included animal studies

First author, year of publication	POI induction model	Number of animals	Breed	Age (weeks)	Number of administration	Administered volume per ovary (µl)
Cetin C et al., 2024 [71]	chemical	6	Sprague–Dawley	N.A	3	200
Allam EA et al., 2022 [72]	ischaemia	10	Wistar albino	N.A	1	900
Bostancı MS et al*.,* 2022 [73]	ischaemia	8	Sprague–Dawley albino	N.A	1	500
Budak Ö et al., 2022 [74]	chemical	7	Sprague–Dawley	9–11	3	200
Shamrai VA et al., 2022 [75]	chemical	5	Wistar	< 52	3	200
Ahmadian S et al., 2020 [76]	chemical	30	Wistar albino	9–10	1	10
Bahmanpour S et al*.*, 2020 [77]	chemical	21	BALB/c^a^	8	1 + 6	220
El Bakly W et al*.*, 2020 [78]	galactose	10	Sprague–Dawley	6	4	50
Ozcan P et al*.,* 2020 [7]	chemical	8	Sprague–Dawley	9–11	3	200
Huang Q et al*.*, 2019 [79]	chemical	6	Sprague–Dawley	8–10	1	30
Vural B et al*.*, 2019 [80]	chemical	12	Fisher F344 inbreed	6	1	30

^a^In this study, the animals were mice instead of rats

### Laboratory parameters

#### AMH

**Fig. 2:**
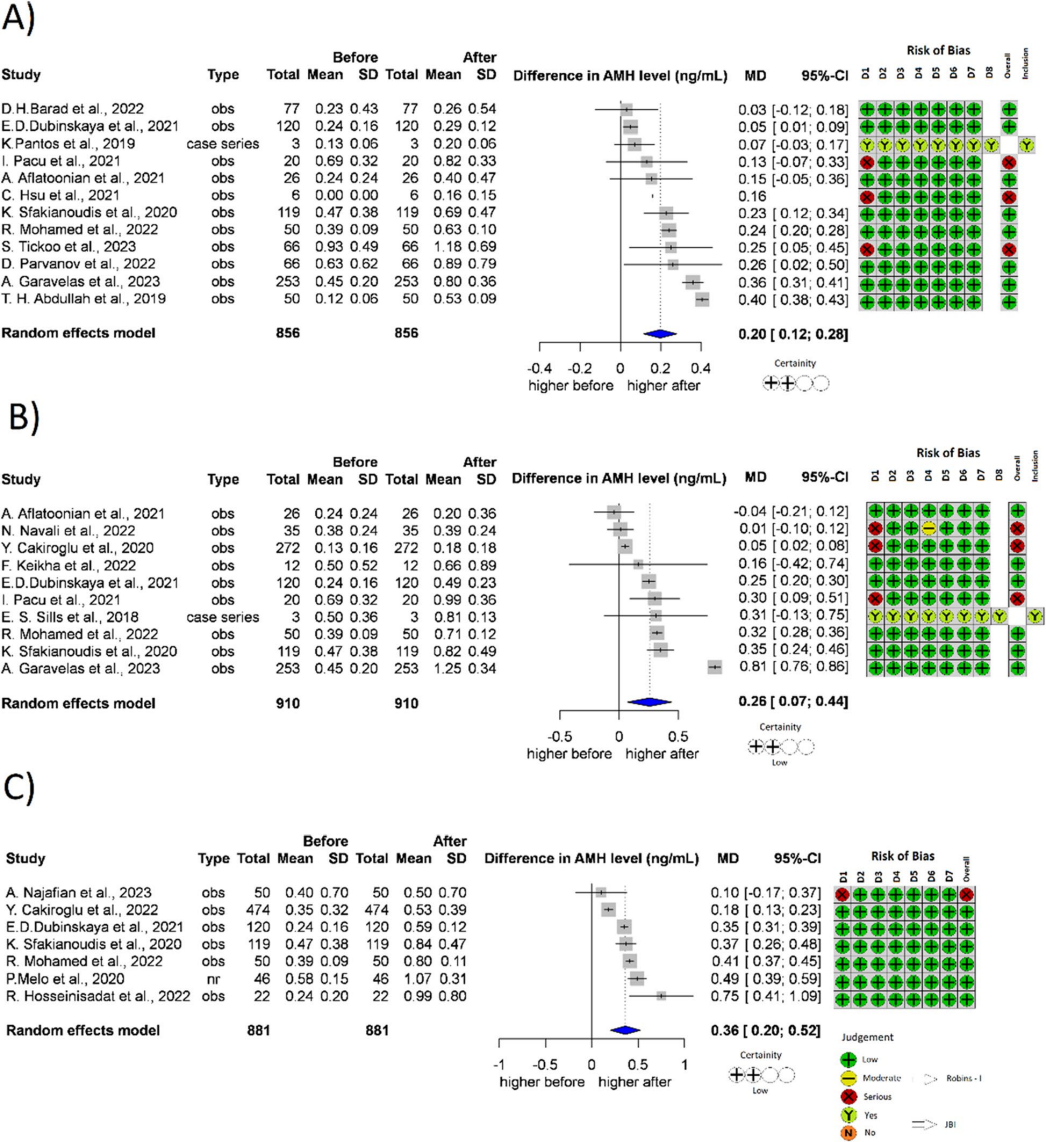
Forest plot of AMH levels before- and after-treatment with PRP. **2A** AMH level one month after the PRP. **2B** AMH level 2 months after the PRP. **2C** AMH level 3 months after the PRP. AMH Anti-Mullerian hormone, CI Confidence interval, SD Standard deviation, CT Controlled trial, obs Observational study, D1-8 Domain 1–8, JBI JBI Manual for Evidence Synthesis, ROBINS-I Risk Of Bias In Non-randomized Studies—of Interventions, SD Standard deviation. *Contains data which was measured after one or three months after the PRP treatment as well

A total of 12 studies [22, 36, 40, 41, 44, 46, 48, 49, 51, 52, 57, 60] (*n* = 856) reported AMH levels recorded one month after treatment (Fig. 2A); 10 studies [22, 33, 36, 44, 45, 48, 49, 52, 53, 61] (*n* = 910) reported AMH levels recorded two months after treatment (Fig. 2B); 7 studies [22, 37, 42–44, 49, 54] (*n* = 881) reported AMH levels recorded three months after treatment (Fig. 2C). In every month after treatment with PRP, the AMH levels significantly increased. One month after the PRP treatment it became 0.20 ng/ml higher than the baseline value (*p* < 0.001, 95% CI: [0.12;0.28]). Two months after the PRP treatment it became 0.26 ng/ml higher than the baseline value (*p* = 0.013, 95% CI: [0.07;0.44]), and after 3 months it became 0.36 ng/ml higher (*p* = 0.002, 95% CI: [0.20;0.52]).

#### FSH

**Fig. 3:**
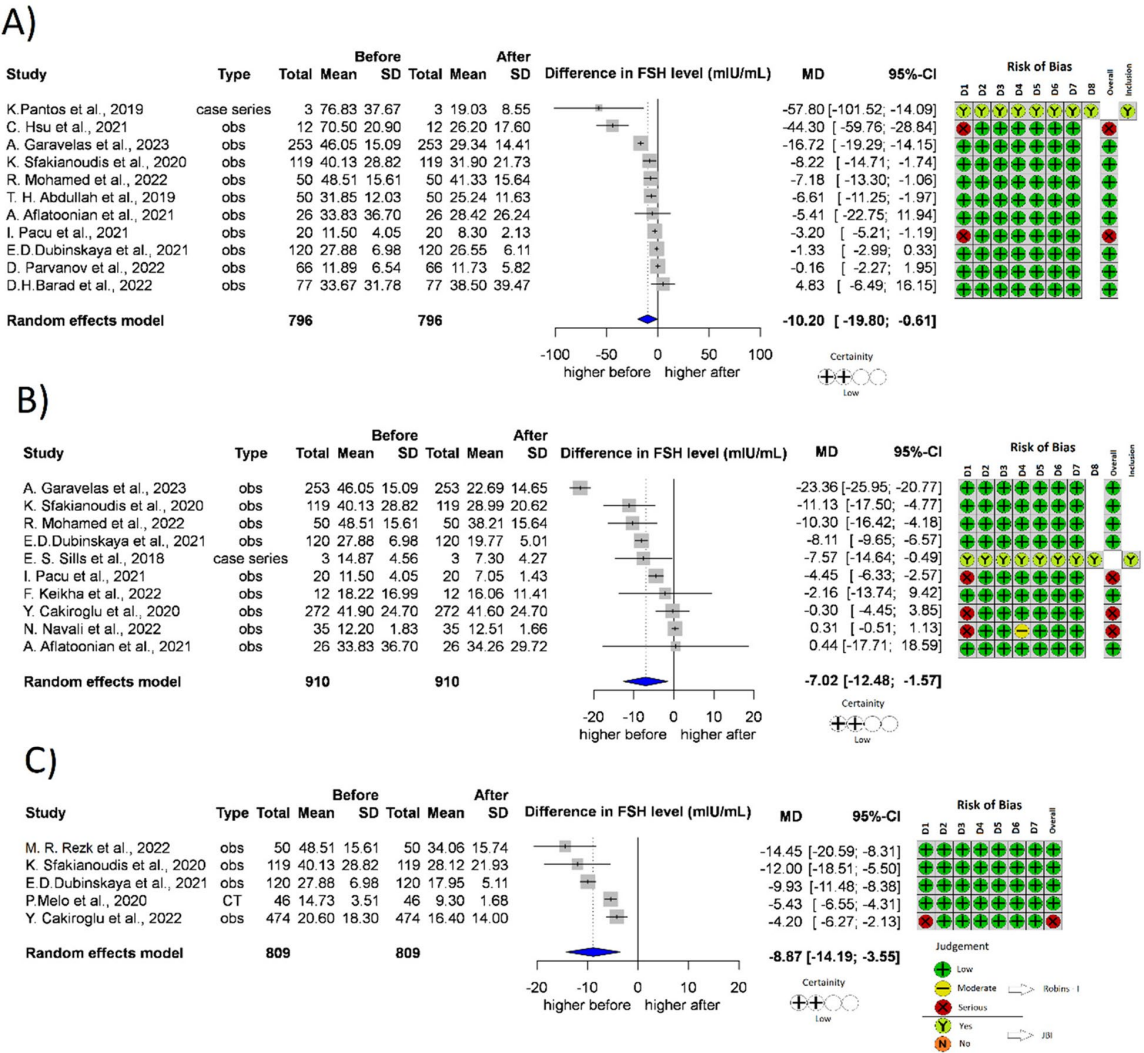
Forest plot of FSH levels before- and after-treatment with PRP. **3A** FSH level one month after the PRP. **3B** FSH level 2 months after the PRP. **3C** FSH level 3 months after the PRP. FSH Follicle-stimulating hormone, CI Confidence interval, SD Standard deviation, CT Controlled trial, obs Observational study, D1-8 Domain 1–8, JBI JBI Manual for Evidence Synthesis, ROBINS-I Risk Of Bias In Non-randomized Studies—of Interventions. *Contains data which was measured after one or three months after the PRP treatment as well

A total of 11 studies [22, 36, 41, 44, 46, 48, 49, 51, 52, 57, 60] (*n* = 796) reported FSH levels recorded one month after treatment (Fig. 3A); 10 studies [22, 33, 36, 44, 45, 48, 49, 52, 53, 61] (*n* = 910) reported FSH levels recorded two months after treatment (Fig. 3B); 5 studies [22, 42, 44, 49, 54] (*n* = 809) reported FSH levels three months after treatment (Fig. 3C). Analysing the FSH values recorded one month after the treatment, the mean difference was as high as -10.20 mIU/ml (*p* = 0.039, 95% CI:[-19.8;-0.61]). After 2 months, the mean difference was -7.02 mIU/mL (*p* = 0.017, 95% CI: [-12.48;-1.57]), after 3 months, the mean difference was -8.87 mIU/mL (*p* = 0.010, 95% CI: [-14.19;-3.55]). In every measured month after treatment with PRP, the FSH levels significantly decreased.

#### LH

A total of 6 studies [22, 36, 48, 51, 52, 60] (*n* = 414) reported LH levels recorded one month after treatment (Fig. S1A); 5 studies [22, 36, 45, 48, 52] (*n* = 434) reported two months after treatment (Fig. S1B).

In every month after treatment with PRP, the LH levels decreased, but it did not reach the level of significance. One month after the PRP treatment, the level of LH was lower with 7.55 mIU/ml than the baseline value (*p* = 0.126, 95% CI: [-18.15;3.05]). Two months after the PRP treatment, the level of LH was lower with 3.28 mIU/ml than the baseline value (*p* = 0.133, 95% CI: [-8.17;1.60]).

##### Estradiol

**Fig. 4:**
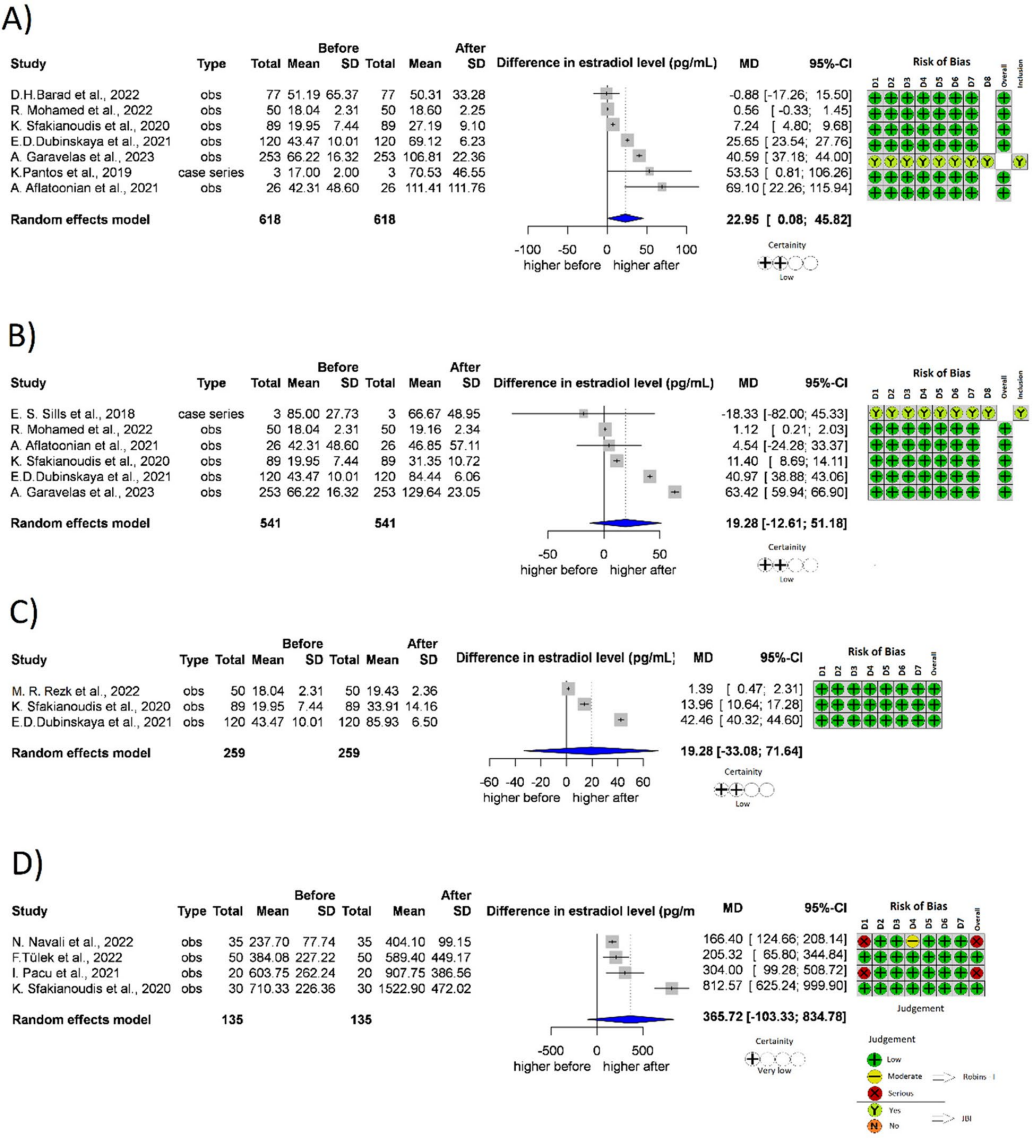
Forest plot of Estradiol levels before- and after-treatment with PRP. **4A** Estradiol level one month after the PRP. **4B** Estradiol level 2 months after the PRP. **4C** Estradiol level 3 months after the PRP. **4D** Mid-cycle estradiol level. CI Confidence interval, SD Standard deviation, CT Controlled trial, obs Observational study, D1-8 Domain 1–8, JBI JBI Manual for Evidence Synthesis, ROBINS-I Risk Of Bias In Non-randomized Studies—of Interventions. *Contains data which was measured after one or three months after the PRP treatment as well

A total of 7 studies [22, 36, 41, 44, 48, 49, 60] (*n* = 618) reported Estradiol levels measured one month after treatment (Fig. 4A); 6 studies [22, 36, 44, 48, 49, 61] (*n* = 541) reported two months after treatment (Fig. 4B); 3 studies [22, 44, 49] (*n* = 259) reported three months after treatment (Fig. 4C). In every month after treatment with PRP, the Estradiol levels increased compared to the baseline value, the difference was significant in the first month, but did not reach the level of significance in the second and third months. After 1 month, the mean difference was 22.95 pg/ml (*p* = 0.049, 95% CI: [0.08; 45.82]). After 2 months, the mean difference was 29.28 pg/ml (*p* = 0.362, 95% CI: [-12.61; 51.18]), after 3 months, the mean difference was 19.28 pg/ml (*p* = 0.254, 95% CI: [-33.08;71,64]).

In terms of mid-cycle estradiol level after the PRP treatment, 4 studies were included [22, 45, 47, 52] with 135 patients treated (Fig. 4D). The mid-cycle estradiol level after the PRP treatment was higher than the baseline value, with 365.72 pg/ml, but the difference was not significant (*p* = 0.089, 95% CI: [-103.33;834.78]).

### IVF treatment outcomes

We examined 3 main parameters characterizing the dynamics of IVF treatments: the AFC, the number of retrieved oocytes, and the number of embryos created.

**Fig. 5:**
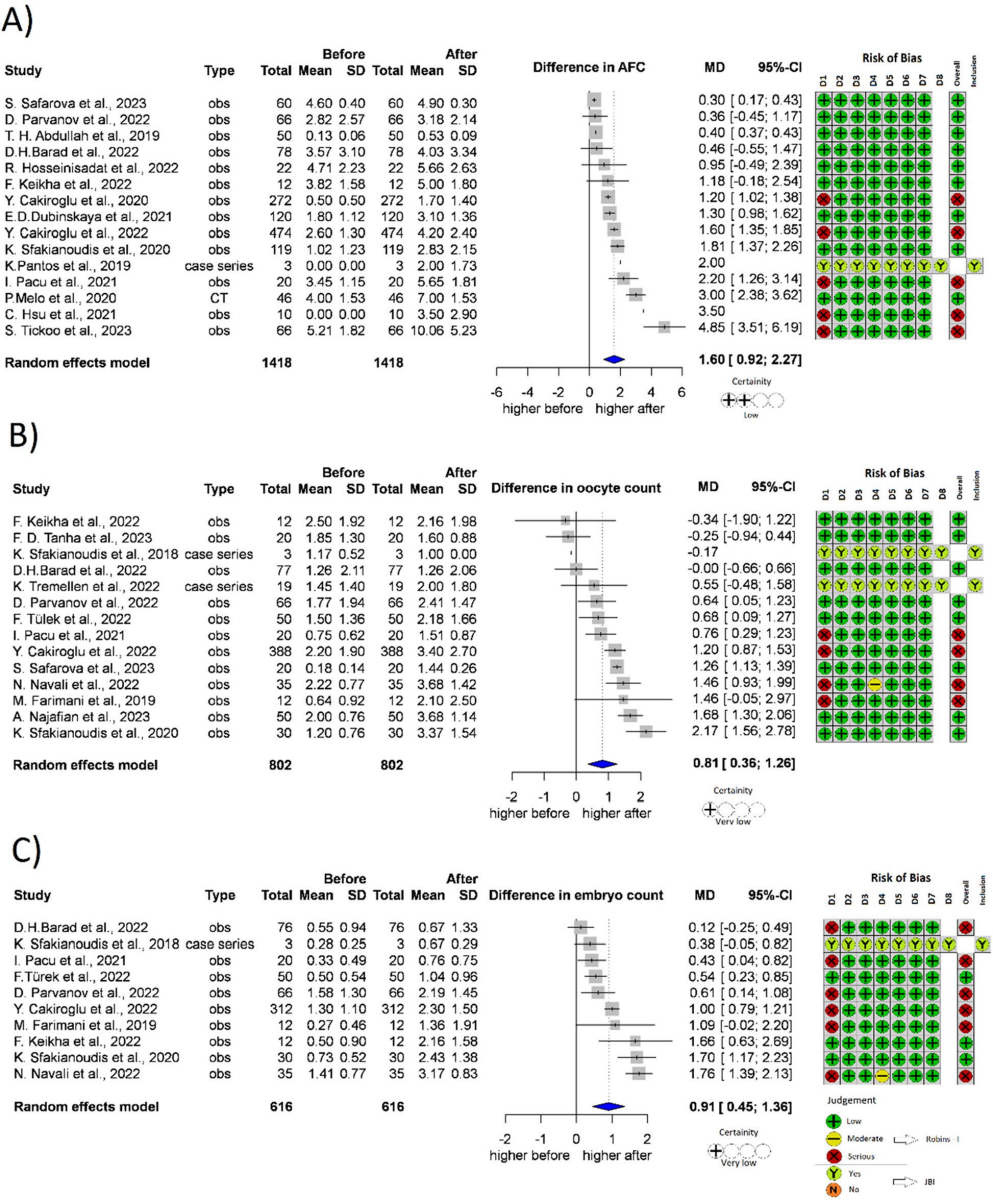
Forest plot of fertility parameters before- and after-treatment with PRP. **5A** AFC level after the PRP. **5B** oocyte count after the PRP. **5C** embryo count after the PRP. AFC antral follicle count, CI Confidence interval, SD Standard deviation, CT Controlled trial, obs Observational study, D1-8 Domain 1–8, JBI JBI Manual for Evidence Synthesis, ROBINS-I Risk Of Bias In Non-randomized Studies—of Interventions

In terms of AFC, 15 studies were included [22, 33, 38, 40–43, 46, 49, 51–54, 57, 60] with 1418 patients treated (Fig. 5A). The post-treatment antral follicle count was higher with 1.60 follicles than the baseline, and the difference was significant (*p* =  < 0.001, 95% CI: [0.92; 2.27]).

A total of 14 studies [6, 22, 33, 37–39, 41, 42, 45–47, 52, 58, 59] (*n* = 802) reported retrieved oocyte count after PRP treatment (Fig. 5B); the mean difference was significant, 0.81 more oocytes were retrieved (*p* = 0.002, 95% CI: [0.36; 1.26]).

In terms of embryo count, 10 studies were included [6, 22, 33, 41, 42, 45–47, 50, 52] with 616 patients treated (Fig. 5C). The post treatment embryo count was 0.91 embryo higher, than the baseline value. The difference was significant (*p* = 0.001, 95% CI: [0.45;1.36]).

### Pregnancies, Livebirths

**Fig. 6:**
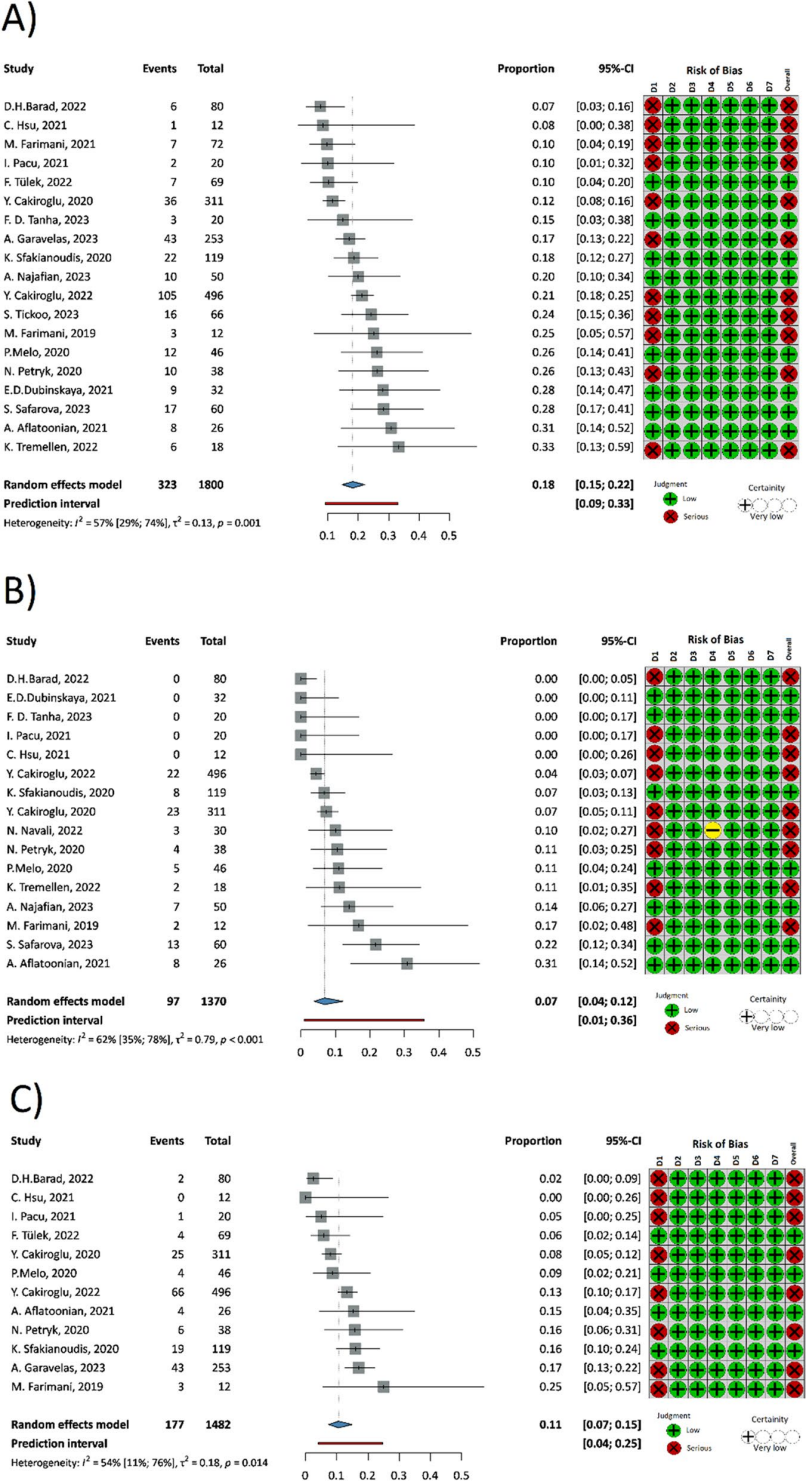
Forest plot of Pregnancies and Livebirth after-treatment with PRP. **6A** Biochemical pregnancies after the PRP. **6B** Spontaneous pregnancies after the PRP. **6C** Livebirths after the PRP. CI Confidence interval, D1-8 Domain 1–8

In terms of biochemical pregnancy, we were able to include 19 studies [22, 36–42, 47–55, 58, 59] with 1800 patients treated. (Fig. 6A) 323 of these were able to reach a biochemical pregnancy. Meta-analysis of incidence of biochemical pregnancy following PRP treatment showed a rate with a proportion of 0.18 (95% CI: 0.15–0.22).

A total of 16 studies [22, 37–39, 41, 42, 45, 48, 49, 51–55, 58, 59] (*n* = 1370) reported spontaneous pregnancy after PRP treatment. (Fig. 6B) 97 patients were able to reach a pregnancy without any hormonal treatment. Meta-analysis of incidence of spontaneous pregnancy following PRP treatment showed rate with a proportion of 0.07 (95% CI: 0.04–0.12).

In the case of livebirth, 12 studies were included [22, 36, 41, 42, 47, 48, 51–55, 58] with 1482 patients treated (Fig. 6C). 177 of these were able to deliver a healthy baby. Meta-analysis of incidence of livebirth following PRP treatment showed a rate with a proportion of 0.11 (95% CI: 0.07–0.15).

### Animal studies

Animal studies were meta-analyzed separately from human studies. In contrast to human studies, animal experiments did not lead to clearly positive results. Despite the fact that all of the included studies were randomized controlled trials by study design, they were characterized by a very low number of items and a very high degree of heterogeneity in the preparation, use, and administration of PRP. We were able to meta-analyze the following outcomes from these studies: AMH, FSH, Estradiol. The results of our analyses are found in the Supplementary Fig. 2.

### Risk of bias and GRADE assessment

The risk of bias assessment highlighted several issues, mainly the absence of any information about the male factor, while the unknown BMI of the patients introduced a serious confounding factor to several studies. Another problem was found in the different methods of PRP treatment and the IVF treatments that followed them. In the case of PRP, the method of preparation, the number of administrations, and the administered volume were heterogenous. The IVF treatments were highly heterogenous in terms of the followed treatment protocol. The time between the administration of PRP to the ovaries and the IVF cycle was also variable, and in some cases it was unknown. Therefore, the interpretation of these results was complicated with regards to judging the effect of PRP treatment of the ovaries. Cakiroglu et al. [53] provided a summary of the hormone test results with different time interval measurements; thus, their data was calculated for the second month results during the quantitative synthesis, based on the largest number of samples.

In most outcomes, the GRADE assessment result was “low quality”, because only observational studies were included in the analysis. Lack of separate control groups, and randomization means a serious limitation. In some cases, downgrading was needed, because of high risk of bias.

## Discussion

In our meta-analysis, we examined the change of hormone levels one, two, and three months after PRP treatment of the ovary. It is noteworthy that the cited articles did not disclose hormone levels from all three months in every case; therefore, the hormone levels measured in different months originate from the work of various authors.

Consequently, the comparison of individual months can only be interpreted with caution, and this comparison alone is not suitable for determining the ideal start time of the IVF cycle after PRP treatment. Regardless, it is a significant result that in the case of AMH, while the hormone level increased significantly in all three months, the highest increase was registered in the third month, thus providing a clue for the optimal timing of starting reproductive treatment. The results suggest that PRP treatment may improve the rejuvenation of the ovary within a month, and its effect is sustained on a significant level for at least three months. Most authors have not examined PRP treatment in a time interval longer than three months; therefore, there is no information on how long the positive effect of PRP treatment lasts.

AMH is a substantial prognostic factor regarding live birth. According to Reijnders et al., the level of AMH is a sufficient factor on its own for predicting the success of IVF treatments [81].

FSH levels are inversely proportional to fertility. Increased levels of the hormone suggest the depletion of the ovary, and predict a poor success rate for assisted reproductive treatments [82]. Based on our analysis, the FSH levels decreased significantly in all three months they were examined after PRP treatment. The decrease of the FSH level reached 7–11 IU/I.

An elevated FSH value has an unfavorable effect on the oocyte quality. Based on the publication by dos Santos et al., the granular changes observed in the cytoplasm and the presence of vacuoles are significantly more frequent in the oocytes aspirated from patients with increased level of FSH. Thus, the PRP treatment can improve the oocyte quality through the long-term reduction of the FSH value [83].

On the other hand, many clinical protocols decide whether a patient can be treated based on the FSH value. This limit value is typically between 15–20 IU/l, where assisted reproductive treatment is not started in patients with FSH values that exceed this. As a result of PRP treatment, many patients who previously proved unsuitable due to their elevated FSH values can participate in assisted reproduction treatment thanks to the decreasing FSH value.

Regarding the examination of LH values, we found a parallel decreasing trend with FSH after PRP treatment, but this level did not reach the level of significance.

The estradiol levels increased in all three months of the study, but its degree reach the level of statistical significance only in the first month.

On the other hand, the estradiol value in the middle of the cycle, which shows a correlation with the follicular maturation, showed a remarkable elevation; however, it did not reach the level of statistical significance.

AFC is one of the most important markers of ovarian reserve and its ability to be stimulated in a given cycle. After PRP treatment, AFC increased by a bit more than one and a half (1.60) antral follicles, which represents a significant improvement.

As a result, the number of aspirated oocytes increased by 0.81 and the number of created embryos by almost the same amount (0.91), which evidences a significant improvement in both cases.

Analyzing the data of 1370 patients in this patient population with an extremely poor reproductive prognosis, we detected a spontaneous pregnancy rate of 7%. This is extremely important because it proves that PRP treatment can be an effective treatment resulting in successful pregnancy even for those patients who refuse any kind of assisted reproduction, for example, based on religious or ethical reasons.

After PRP treatment, rates of 18% of clinical pregnancy and 11% of livebirth were achieved in the patient population, where the use of donor oocytes is typically recommended. In many cases, the analyzed publications did not follow up all clinical pregnancies to their end [53, 55], so the real live birth rate may be even higher in the future.

None of the analyzed 38 human publications mentioned any complication in connection with PRP treatments, which evidences the safety of the method. Since the introduction of intraovarian PRP treatment, the methodology has also significantly developed and has been favorably steered in the direction of micro-invasiveness. Treatments previously carried out by laparoscopy are now routinely performed with the guidance of vaginal ultrasound, in a manner completely similar to oocyte retrieval, with the same instrumentation and by professionals skilled in it.

### Former studies of the field

PRP treatment for ovarian rejuvenation was first brought into discussion by Pantos and colleagues, in a short communication at the ESHRE Annual Meeting in 2016. The menstruation cycle was restored in a case series of 8 perimenopausal women after PRP was injected directly into their ovaries [21].

Consecutive observational studies with increasing sample sizes were published in 2020, one of which was conducted by Sills et al., and reported contradictory results. Although some patients in this study benefited from the treatment in terms of improved hormone levels, the null hypothesis could not be rejected for the primary outcome, which was that PRP does not improve hormone levels. However, the authors concluded that the treatment was safe, as no adverse events were associated with the intervention observed [56].

In the same year, Cakiroglu et al. conducted a study wherein 311 women were administered PRP alongside the IVF treatment. 23 women achieved spontaneous pregnancy and an overall improvement in atrial follicle count was observed as well an increase in their AMH levels [53].

Another study involving 119 women was conducted in 2020, in which a majority of the participants with premature ovarian insufficiency were observed to have improved hormone levels. Similar outcomes were reported in the perimenopausal group. In all groups of participants, the number of spontaneous pregnancies and live births were improved [22].

The first systematic review in this topic was conducted by Panda et al. in 2020, wherein the authors stated the promising results of ovarian PRP treatment. They concluded that intra-ovarian autologous PRP infusion increases the ovarian reserve parameters resulting in increased mature oocyte yield and fertilization rate, as well as the formation of good quality embryos. However, due to the lack of adequate clinical evidence at that time, performing a meta-analysis was not possible [84].

The first randomized controlled trial in this topic was recently published by Barrenetxea et al. This RCT could analyze just a small number of patients (30 patients on the treated arm). As a result, they could verify, that ovarian PRP therapy increases the number of retrieved oocytes. but they can not observe any significant increase in embryo quality of developed blastocysts [85].

We have found just 3 meta-analyses until now, published in 2023 [86–88]. In our meta-analysis we found three to four as many publications appropriate for inclusion compared to the published papers so our patient number became much higher and during the statistical analysis resulted more significant results on the analysed much wider parameters. Given the significantly higher number of patients, we were able to separately analyse the hormonal results 1, 2 and 3 months after the PRP treatment.

### Strengths and limitations

The main strength of this study was the protocolized, systematic approach to this relatively new topic, adheres to all Cochrane Collaboration guidelines and a registered protocol. We can state, that our present research is based on the far largest patient number in the subject, and the most comprehensive analysis, able to prove the most significant changes.

A significant part of the studies published on the subject involved only a small number of cases. Through our meta-analysis, however, we were able to collect such a high number of cases in total, that we were able to verify significant changes in several important fertility parameters as a result of PRP treatment.

The main limitation in our study was the lack of adequately designed clinical literature. Apart from the single RCT with a limited number of cases, our findings were based solely on observational studies. Analyses of several outcomes, such as serum LH and estradiol, come from pooling low sample size studies. Another important limitation is the different methodologies employed by the various centers participating in the research, with respect to preparing and applying the PRP, which makes it difficult to generalize the results and requires the creation of a consensus among experts in the field. The comparability of the results of IVF treatments following PRP also presented difficulties due to their extreme methodological heterogeneity.

### Implications for practice and research

In our opinion, our meta-analysis credibly proves the raison d'être of ovarian PRP treatment in patients with reduced ovarian reserve. This evidence paves the way for a multicenter, randomized study, with a large patient number, and a longer follow-up period, which should unify the exact method of optimal preparation and application of PRP and the most effective methodology of subsequent reproductive interventions. Further research is needed on the optimal method of PRP preparation, the most effective way of administering PRP, the optimal timing of the start of assisted reproductive treatment after PRP, and how long the results of PRP treatment last. An RCT with a large number of cases is also needed, based on the lessons we mentioned earlier in connection with the critical analysis of the analyzed articles. Based on all this, a standardized, effective, and safe protocol can make ovarian PRP an important adjuvant in the future for the reproductive treatment of women who desire to have children, but are affected by diminished ovarian reserve or premature ovarian insufficiency [89].

## Conclusions

Based on the analysis of most of the cases to date, which come from predominantly observational studies of variable quality, PRP treatment of the ovaries appears to be a suitable and effective procedure for improving several key fertility parameters in women with low ovarian reserve.

### Supplementary Information

**Additional file 1:** **Fig. S1**. Forest plot of LH levels before- and after-treatment with PRP. S1A LH level one month after the PRP. S2B LH level 2 months after the PRP. **Fig. S2.** Forest plot of FSH, AMH and Estradiol levels before- and after-treatment with PRP in animal studies. S2A Animal AMH level after the PRP. S2B Animal FSH level after the PRP. S2C Animal Estradiol level after the PRP. **Table S1.** PRISMA Checklist of reporting items. **Table S2.** Statistical data before the correction.

## Data Availability

No datasets were generated or analysed during the current study.

## Abbreviations

AMH: Anti-Müllerian Hormone
CI: Confidence interval
GRADE: Grades of Recommendation, Assessment, Development and Evaluation framework
FSH: Follicle Stimulating Hormone
IVF: In vitro fertilization
LH: Lutheinizing Hormone
MD: Mean Difference
VEGF: Vascular endothelial growth factor
PDGF: Platelet-derived growth factor
IGF: Insulin-like growth factor
FGF: Fibroblast growth factor
TGF-β: Transforming growth factor-β
BMI: Body mass index
