# Supplementary material for: Platelet-rich plasma (PRP) treatment of the ovaries significantly improves fertility parameters and reproductive outcomes in diminished ovarian reserve patients: a systematic review and meta-analysis
Source: J Ovarian Res. 2024 May 17;17:104. doi: 10.1186/s13048-024-01423-2 (PMC11100055; doi:10.1186/s13048-024-01423-2)
Supplement: Supplementary file 1 — Additional file 1: Fig. S1. Forest plot of LH levels before- and after-treatment with PRP. S1A LH level one month after the PRP. S2B LH level 2 months after the PRP. Fig. S2. Forest plot of FSH, AMH and Estradiol levels before- and after-treatment with PRP in animal studies. S2A Animal AMH level after the PRP. S2B Animal FSH level after the PRP. S2C Animal Estradiol level after the PRP. Table S1. PRISMA Checklist of reporting items. Table S2. Statistical data before the correction. [file 13048_2024_1423_MOESM1_ESM.docx]

**Platelet-Rich Plasma (PRP) Treatment of the Ovaries Significantly Improves Fertility Parameters and Reproductive Outcomes in Diminished Ovarian Reserve Patients: A Systematic Review and Meta-Analysis**

Running title: PRP Treatment of the Ovaries: A Meta-Analysis

Máté Éliás^1^, Márton Kónya^1^, Zsófia Kekk^1^, Caner Turan^1^, Isabel Pinto Amorim das Virgens^1^, Réka Tóth^1^, Márton Keszthelyi^1,2^, Péter Hegyi^1,3,4^, Szabolcs Várbíró^1^, Miklós Sipos^1,2^

**Affiliations:** 1: Centre for Translational Medicine, Semmelweis University, Budapest, Hungary; 2: Department of Obstetrics and Gynecology, Semmelweis University, Budapest, Hungary; 3: Division of Pancreatic Diseases, Semmelweis University, Budapest, Hungary; 4: Institute for Translational Medicine, University of Pécs, Pécs, Hungary;

**Corresponding author:** Miklós Sipos MD, PhD: Centre of Assisted Reproduction, Semmelweis University, 78/B Üllői út, Budapest, Hungary, 1083. Phone: +36309545908. E-mail: [sipos.miklos.dr@gmail.com](mailto:sipos.miklos.dr@gmail.com). ORCID: 0009-0009-0827-6689

**Description**

**Supplementary Figure 1.:** Forest plot of LH levels before- and after-treatment with PRP. S1A: LH level one month after the PRP. S2B: LH level 2 months after the PRP.

**Supplementary Figure 2.:** Forest plot of FSH, AMH and Estradiol levels before- and after-treatment with PRP in animal studies. S2A: Animal AMH level after the PRP. S2B: Animal FSH level after the PRP. S2C: Animal Estradiol level after the PRP

**Supplementary Table 1.:** PRISMA checklist of reporting items.

**Supplementary Table 2.:** Statistical data before the corrections


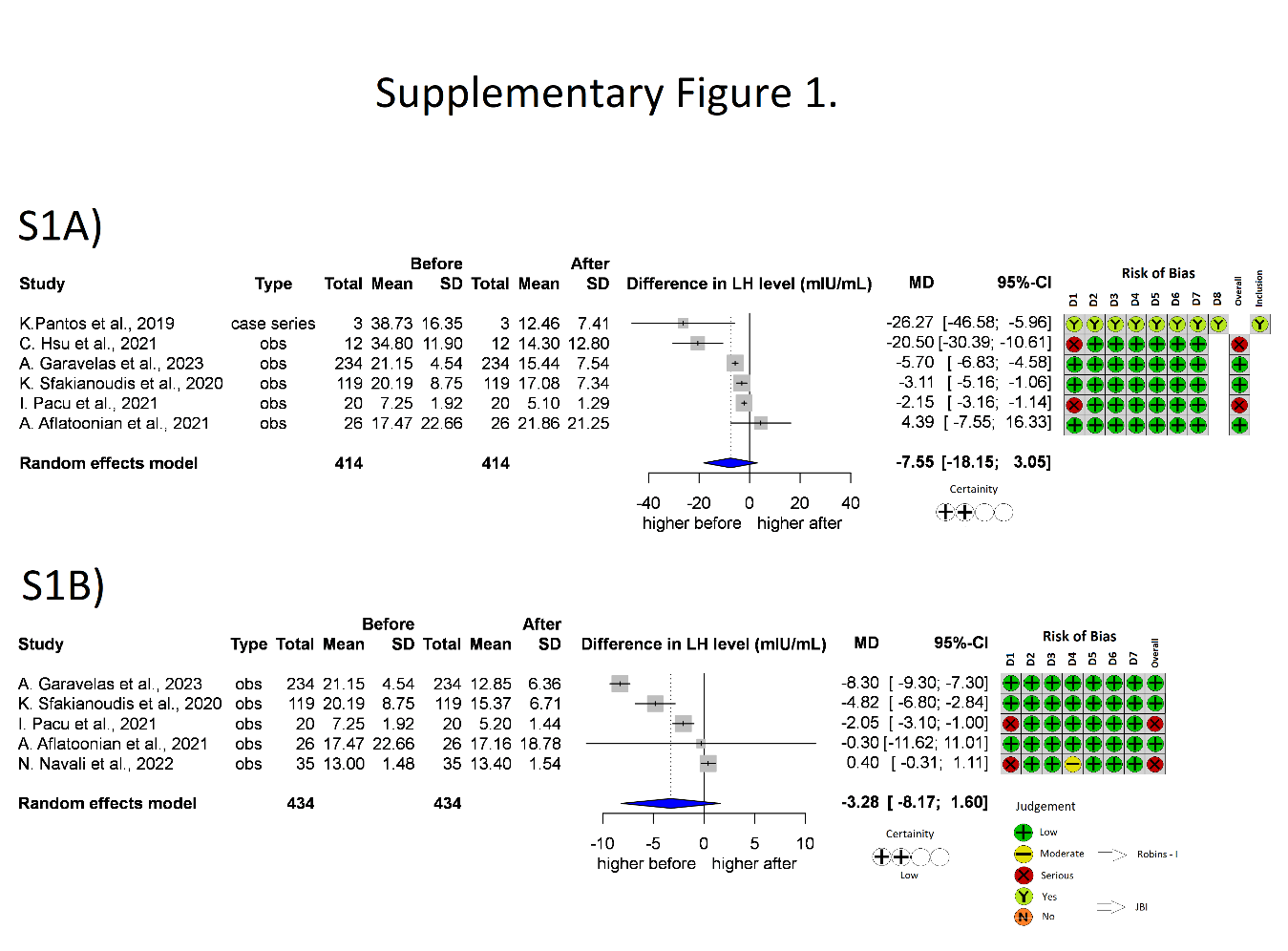


**Supplementary Figure 1.: Forest plot of LH levels before- and after-treatment with PRP.** S1A: LH level one month after the PRP. S2B: LH level 2 months after the PRP. LH: Luteinizing hormone;  CI: confidence interval; SD: standard deviation; obs : observational study; D1-8: Domain 1-8; JBI: JBI Manual for Evidence Synthesis; ROBINS-I: Risk Of Bias In Non-randomised Studies - of Interventions


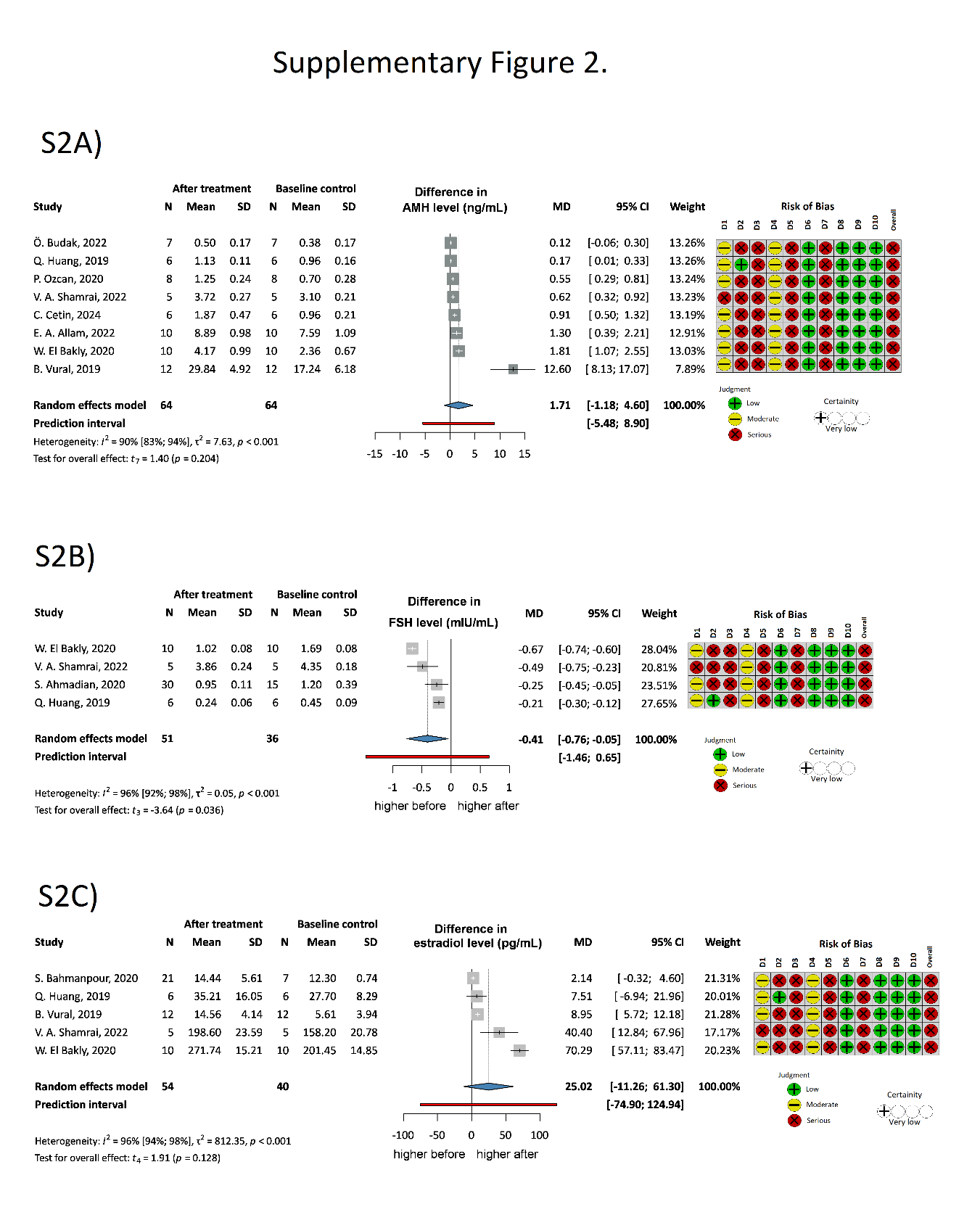


**Supplementary Figure 2.: Forest plot of FSH, AMH and Estradiol levels before- and after-treatment with PRP in animal studies.** S2A: Animal AMH level after the PRP. S2B: Animal FSH level after the PRP. S2C: Animal Estradiol level after the PRP.; AMH: Anti-Mullerian hormone; FSH: Follicle-stimulating hormone; CI: confidence interval; SD: standard deviation; D1-10: Domain 1-10; N: number

**Supplementary Files**

**Table S1.** PRISMA checklist of reporting items.

| **Section and Topic** | **Item #** | **Checklist item** | **Location where item is reported** |
| --- | --- | --- | --- |
| **TITLE** | | |  |
| Title | 1 | Identify the report as a systematic review. | Title |
| **ABSTRACT** | | |  |
| Abstract | 2 | See the PRISMA 2020 for Abstracts checklist. | Abstract |
| **INTRODUCTION** | | |  |
| Rationale | 3 | Describe the rationale for the review in the context of existing knowledge. | Section 1 |
| Objectives | 4 | Provide an explicit statement of the objective(s) or question(s) the review addresses. | Section 1 |
| **METHODS** | | |  |
| Eligibility criteria | 5 | Specify the inclusion and exclusion criteria for the review and how studies were grouped for the syntheses. | Section 2.2 |
| Information sources | 6 | Specify all databases, registers, websites, organisations, reference lists and other sources searched or consulted to identify studies. Specify the date when each source was last searched or consulted. | Section 2.1 |
| Search strategy | 7 | Present the full search strategies for all databases, registers and websites, including any filters and limits used. | Section 2.1 |
| Selection process | 8 | Specify the methods used to decide whether a study met the inclusion criteria of the review, including how many reviewers screened each record and each report retrieved, whether they worked independently, and if applicable, details of automation tools used in the process. | Section 2.3 |
| Data collection process | 9 | Specify the methods used to collect data from reports, including how many reviewers collected data from each report, whether they worked independently, any processes for obtaining or confirming data from study investigators, and if applicable, details of automation tools used in the process. | Section 2.3 |
| Data items | 10a | List and define all outcomes for which data were sought. Specify whether all results that were compatible with each outcome domain in each study were sought (e.g. for all measures, time points, analyses), and if not, the methods used to decide which results to collect. | Section 2.3 |
|  | 10b | List and define all other variables for which data were sought (e.g. participant and intervention characteristics, funding sources). Describe any assumptions made about any missing or unclear information. | Section 2.4 |
| Study risk of bias assessment | 11 | Specify the methods used to assess risk of bias in the included studies, including details of the tool(s) used, how many reviewers assessed each study and whether they worked independently, and if applicable, details of automation tools used in the process. | Section 2.3 |
| Effect measures | 12 | Specify for each outcome the effect measure(s) (e.g. risk ratio, mean difference) used in the synthesis or presentation of results. | Section 2.4 |
| Synthesis methods | 13a | Describe the processes used to decide which studies were eligible for each synthesis (e.g. tabulating the study intervention characteristics and comparing against the planned groups for each synthesis (item #5)). | Section 2.4 |
|  | 13b | Describe any methods required to prepare the data for presentation or synthesis, such as handling of missing summary statistics, or data conversions. | Section 2.4 |
|  | 13c | Describe any methods used to tabulate or visually display results of individual studies and syntheses. | Section 2.4 |
|  | 13d | Describe any methods used to synthesize results and provide a rationale for the choice(s). If meta-analysis was performed, describe the model(s), method(s) to identify the presence and extent of statistical heterogeneity, and software package(s) used. | Section 2.4 |
|  | 13e | Describe any methods used to explore possible causes of heterogeneity among study results (e.g. subgroup analysis, meta-regression). | Section 2.4 |
|  | 13f | Describe any sensitivity analyses conducted to assess robustness of the synthesized results. | Section 2.4 |
| Reporting bias assessment | 14 | Describe any methods used to assess risk of bias due to missing results in a synthesis (arising from reporting biases). | Section 2.4 |
| Certainty assessment | 15 | Describe any methods used to assess certainty (or confidence) in the body of evidence for an outcome. | Section 2.3 |
| **RESULTS** | | |  |
| Study selection | 16a | Describe the results of the search and selection process, from the number of records identified in the search to the number of studies included in the review, ideally using a flow diagram. | Section 3.1, Table 1 |
|  | 16b | Cite studies that might appear to meet the inclusion criteria, but which were excluded, and explain why they were excluded. | Table 1 |
| Study characteristics | 17 | Cite each included study and present its characteristics. | Section 3.1  Table 2 |
| Risk of bias in studies | 18 | Present assessments of risk of bias for each included study. | Figure 1,2,3,4,5 |
| Results of individual studies | 19 | For all outcomes, present, for each study: (a) summary statistics for each group (where appropriate) and (b) an effect estimate and its precision (e.g. confidence/credible interval), ideally using structured tables or plots. | Section 3.2, 3.3,3.4  Figure 1-5. |
| Results of syntheses | 20a | For each synthesis, briefly summarise the characteristics and risk of bias among contributing studies. | Figure 1-5. |
|  | 20b | Present results of all statistical syntheses conducted. If meta-analysis was done, present for each the summary estimate and its precision (e.g. confidence/credible interval) and measures of statistical heterogeneity. If comparing groups, describe the direction of the effect. | Section 3.2, 3.3,3.4  Figure 1-5. |
|  | 20c | Present results of all investigations of possible causes of heterogeneity among study results. | Section 2.4 |
|  | 20d | Present results of all sensitivity analyses conducted to assess the robustness of the synthesized results. | Section 2.4 |
| Reporting biases | 21 | Present assessments of risk of bias due to missing results (arising from reporting biases) for each synthesis assessed. | Section 3.2  Figure 2 |
| Certainty of evidence | 22 | Present assessments of certainty (or confidence) in the body of evidence for each outcome assessed. | Figure1-5. |
| **DISCUSSION** | | |  |
| Discussion | 23a | Provide a general interpretation of the results in the context of other evidence. | Section 4.1 |
|  | 23b | Discuss any limitations of the evidence included in the review. | Section 4.2. |
|  | 23c | Discuss any limitations of the review processes used. | Section 4.2 |
|  | 23d | Discuss implications of the results for practice, policy, and future research. | Section 4.3 |
| **OTHER INFORMATION** | | |  |
| Registration and protocol | 24a | Provide registration information for the review, including register name and registration number, or state that the review was not registered. | Section 2 |
|  | 24b | Indicate where the review protocol can be accessed, or state that a protocol was not prepared. | Section 2 |
|  | 24c | Describe and explain any amendments to information provided at registration or in the protocol. | Section 2 |
| Support | 25 | Describe sources of financial or non-financial support for the review, and the role of the funders or sponsors in the review. | Funding |
| Competing interests | 26 | Declare any competing interests of review authors. | Conflict of interest |
| Availability of data, code and other materials | 27 | Report which of the following are publicly available and where they can be found: template data collection forms; data extracted from included studies; data used for all analyses; analytic code; any other materials used in the review. | Data availability |

**Supplementary Table 2.: Statistical data before the corrections**

| SIMPLEST MODEL (reported) | **before** | **1 month** | **2 months** | **3 months** | |
| --- | --- | --- | --- | --- | --- |
| **longitudinal pool** | 33.69 [18.66; 48.71] | 49.15 [22.06; 76.24] | 58.15 [24.21; 92.08] | 59.65 [25.41; 93.90] |  |
| **longitudinal pool incl. case series** | 38.80 [23.01; 54.59] | 56.30 [32.88; 79.73] | 56.68 [28.11; 85.25] | 56.02 [27.01; 85.04] |  |
| ALTERNATIVE V MATRIX 1* |  |  |  |  |  |
| **longitudinal pool** | 32.99 [17.51; 48.46] | 47.89 [19.97; 75.80] | 56.47 [21.42; 91.52] | 58.00 [22.66; 93.33] |  |
| **longitudinal pool incl. case series** | 39.17 [23.19; 55.15] | 57.51 [33.72; 81.31] | 56.08 [26.91; 85.25] | 55.01 [25.44; 84.58] |  |
| ALTERNATIVE V MATRIX 2** |  |  |  |  |  |
| **longitudinal pool** | 32.69 [17.11; 48.27] | 47.44 [19.41; 75.47] | 55.93 [20.73; 91.12] | 57.38 [21.91; 92.86] |  |
| **longitudinal pool incl. case series** | 39.15 [22.83; 55.47] | 58.63 [34.41; 82.86] | 56.42 [27.15; 85.69] | 54.88 [25.10; 84.66] |  |

*assumes a first order autoregressive correlation structure between 1, 2, and 3 months values, and a correlation of 0.7 between the before value and each of the others, **assumes a first order autoregressive correlation structure between 1, 2, and 3 months values, and a correlations of 0.8, 0.7, and 0.6 between the before value months 1, 2, and 3, respectively. In brackets are the CI.

Method of PRP preparation

International consensus on the protocol for preparing PRP is missing. The basis for the production of PRP is differential gradient centrifugation. Each component of whole blood has a different specific gravity and is separated into layers when centrifuged (73).

There are two different methods of producing PRP: the PRP method and the buffy-coat method. The PRP method uses fresh blood, which is centrifuged in low-speed to separate the red blood cells. The supernatant plasma is then centrifuged at high-speed to obtain the platelet concentrate. The buffy-coat technique utilizes whole blood, prestored at room temperature. It undergoes a high-speed centrifugation to separate it into three layers: red blood cells, platelets and white blood cells, and platelet-poor plasma. Then, the supernatant plasma is removed, and the buffy-coat is separated. This layer undergoes a second low-speed spin to separate the white blood cells, or a leukocyte filter can be used (73).

The speed and the duration of the centrifugation is an important factor in determining the optimal platelet yield. Sabarish *et al*. found that lower spin rates had higher platelet yields, hypothesizing that high rates could cause platelet clumping or disintegration (74).

There are several kits available on the market to prepare PRP with varying concentrations of platelets, and there is no consensus on optimal concentrations of the components either (75).

73. Dhurat R, Sukesh M. Principles and Methods of Preparation of Platelet-Rich Plasma: A Review and Author's Perspective. J Cutan Aesthet Surg. 2014;7(4):189-97.

74. Sabarish R, Lavu V, Rao SR. A Comparison of Platelet Count and Enrichment Percentages in the Platelet Rich Plasma (PRP) Obtained Following Preparation by Three Different Methods. J Clin Diagn Res. 2015;9(2):Zc10-2.

75. Oudelaar BW, Peerbooms JC, Huis In 't Veld R, Vochteloo AJH. Concentrations of Blood Components in Commercial Platelet-Rich Plasma Separation Systems: A Review of the Literature. Am J Sports Med. 2019;47(2):479-87.
